# Methods used to study the oligomeric structure of G-protein-coupled receptors

**DOI:** 10.1042/BSR20160547

**Published:** 2017-04-20

**Authors:** Hui Guo, Su An, Richard Ward, Yang Yang, Ying Liu, Xiao-Xi Guo, Qian Hao, Tian-Rui Xu

**Affiliations:** 1 Faculty of Environmental Science and Engineering, Faculty of Life Science and Technology, Kunming University of Science and Technology, Kunming 650500, Yunnan, China; 2 First People’s Hospital of Yunnan Province, Kunming, Yunnan, China; 3 Molecular Pharmacology Group, Institute of Molecular, Cell and Systems Biology, College of Medical, Veterinary and Life Sciences, University of Glasgow, Glasgow G12 8QQ, Scotland, U.K.

**Keywords:** computational methods, FRET technology, GPCR, oligomerization

## Abstract

G-protein-coupled receptors (GPCRs), which constitute the largest family of cell surface receptors, were originally thought to function as monomers, but are now recognized as being able to act in a wide range of oligomeric states and indeed, it is known that the oligomerization state of a GPCR can modulate its pharmacology and function. A number of experimental techniques have been devised to study GPCR oligomerization including those based upon traditional biochemistry such as blue-native PAGE (BN-PAGE), co-immunoprecipitation (Co-IP) and protein-fragment complementation assays (PCAs), those based upon resonance energy transfer, FRET, time-resolved FRET (TR-FRET), FRET spectrometry and bioluminescence resonance energy transfer (BRET). Those based upon microscopy such as FRAP, total internal reflection fluorescence microscopy (TIRFM), spatial intensity distribution analysis (SpIDA) and various single molecule imaging techniques. Finally with the solution of a growing number of crystal structures, X-ray crystallography must be acknowledged as an important source of discovery in this field. A different, but in many ways complementary approach to the use of more traditional experimental techniques, are those involving computational methods that possess obvious merit in the study of the dynamics of oligomer formation and function. Here, we summarize the latest developments that have been made in the methods used to study GPCR oligomerization and give an overview of their application.

## Introduction

G-protein-coupled receptors (GPCRs), also called seven transmembrane (TM) receptors or heptahelical receptors, are involved in the regulation of almost all physiological processes [1]. GPCRs constitute the largest family of signalling proteins and are encoded by 3–5% of all genes in animals [2]. A diverse array of molecules (such as ions, nucleotides, amino acids, amines and peptides) or even photons can activate GPCRs and initiate a cascade of signalling events leading to physiological reactions. GPCRs are the targets for nearly 50% of currently marketed drugs [3] and are a major focus in drug development [1]. GPCRs possess a structurally homologous core of seven TM α-helices, which are accompanied by significant variation in the size and structure of the extracellular and intracellular loops [4]. The life cycle of each receptor includes many stages: synthesis, quality control in the endoplasmic reticulum, maturation in the Golgi, delivery to the plasma membrane, ligand interaction and subsequent signalling, endocytosis and subsequent sorting in endosomes resulting in either recycling to the membrane or destruction [2].

GPCRs have been shown to exist or function as monomers, dimers and/or higher order oligomeric complexes including homodimers/oligomers and heterodimers/oligomers. Different GPCR subtypes, even the same receptor at different stages of its life cycle, may exist in different oligomerization states, from monomers to dimers and possibly higher order oligomers [5–10]. The earliest report of a GPCR dimer was that of the formation of a heterodimer between the α2c-adrenergic and the M_3_ muscarinic acetylcholine receptors (M_3_R), which may involve the intermolecular exchange of N- and C-terminal receptor domains [6]. A large number of other GPCR dimers have been identified since then, for example the angiotensin II receptor type 1 (AT_1_R) can associate with the bradykinin receptor B_2_ [11], whereas, β_2_-adrenergic receptor (β_2_-AR) [12], the metabotropic glutamate receptor (mGluR) subtype 5 (mGluR_5_) [13] and rhodopsin can exit as homodimers [14]. Furthermore, several studies have reported the existence of higher ordered GPCR oligomers, examples of this include the μ-opioid receptor (MOR) that can form heterooligomers with the δ-opioid receptor (DOR) and also the β_2_-AR and the cannabinoid receptor type 1 (CB_1_R) [15–17]. The most well-known example of GPCR oligomerization might be the GABAB (γ-aminobutyric acid) receptor with two separate seven TM-spanning subunits, which require the presence of both subunits and the heterodimer is the functional unit in the trafficking of the receptor to the cell membrane [18,19].

GPCR oligomerization has been implicated in some diseases. Pre-eclampsia has been identified as the first disorder known to be associated with altered GPCR heterodimerization, in this case of AT_1_ and B_2_ receptors [20]. In the case of early onset of obesity, mutations in the melanocortin-4 receptor (MC_4_R) (*D90N*) gene are the most frequent monogenic causes of severe obesity and it has dominant negative effect on the WT-MC_4_R/D90N receptor heterodimer [21].

**Figure 1:**
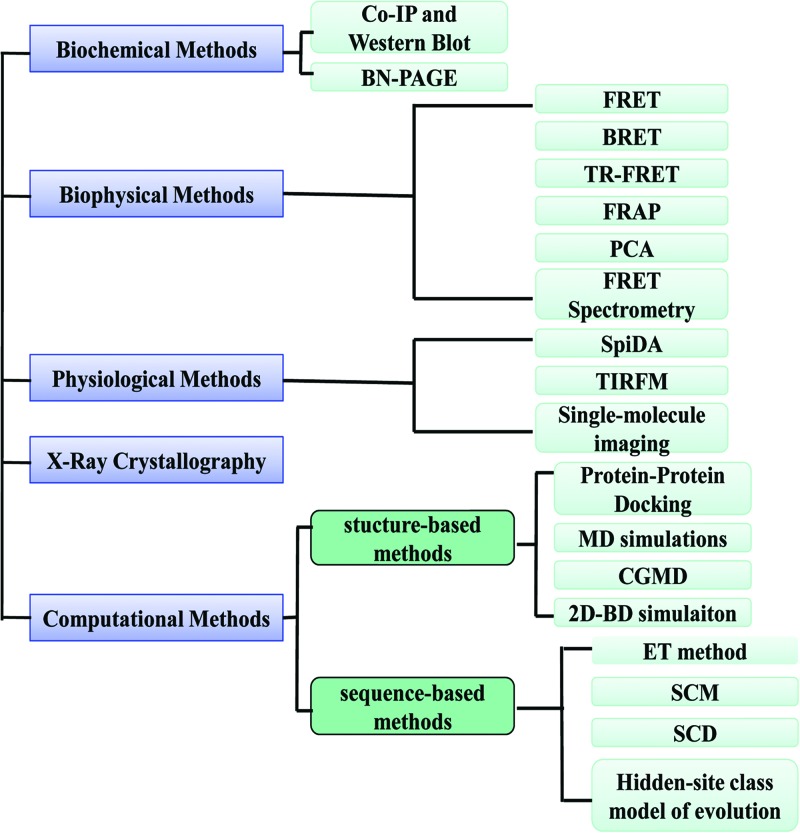
Techniques for studying oligomerization of GPCRs The techniques used to study oligomerization of GPCRs may be divided into five categories, which are biochemical methods, biophysical methods, physiological methods, X-ray crystallography and computational methods. Biochemical approaches include Co-IP and BN-PAGE. Biophysical approaches include FRET, bioluminescence resonance energy transfer (BRET), protein-fragment complementation assays (PCA). SpIDA, single fluorescent-molecule and TIRFM are physiological methods.

Currently, a wide range of methods are available for studying GCPR oligomers (Figure 1) and it should be noted that the strength of the evidence supporting the formation in living cells of a particular GPCR dimer can vary substantially depending on the method used [22]. The use of co-immunoprecipitation (Co-IP) followed by Western blotting is one of the first techniques that provided direct evidence for GPCR oligomerization, although this must be set against its requirement for cell disruption and membrane solubilization [12]. Similarly, the technique of blue-native PAGE (BN-PAGE) is a good choice to show that GPCRs exist as monomers, dimers or higher order oligomers, though it too requires cell disruption and membrane solubilization and it should be noted that it is under the conditions designed to preserve oligomeric structure [23]. Subsequently, the widespread use of biophysical techniques such as resonance energy transfer, fluorescence complementation or a combination of these techniques [24] has resulted in a wealth of information supporting the existence of GPCR oligomers in intact cells [25]. Microscopy-based techniques, such as spatial intensity distribution analysis (SpIDA), total internal reflection fluorescence microscopy (TIRFM) and single-molecule imaging can provide data on dynamics of GPCR oligomers [26]. An intriguing set of results in the field of GPCR oligomerization has been based on previously obtained high-resolution crystallographic structures, including those of the chemokine CXCR4, μ- and κ-opioid receptors, the β1-adrenoceptor and the smoothened receptor [27–30]. Based on the experimental information, computational methods have recently been devised that can mimic the formation of dimers and oligomers and facilitate the study of dimeric and oligomeric interfaces.

In this article, we provide an overview of the methods that have been used to obtain evidence for the existence and function of, at least some, GPCRs in the form of oligomeric units. Currently, one definitive method does not exist and each available method presents various advantages and disadvantages depending upon its characteristics.

## Methods to study oligomers of GPCRs

### Biochemical methods

#### Co-IP and Western blotting

Co-IP and Western blotting are probably one of the first techniques that provided direct evidence for β_2_-AR that it can form homodimers [12].

It has been used to detect oligomeric complexes of a number of GPCRs, such as β_2_-AR [12], DOR [31] and mGluR_5_ [15] receptors. Using epitope tags and appropriate antibodies, the β_2_-AR was found to form homodimers and furthermore, these could be stabilized by agonists, whereas inverse agonists treatment was found to stabilize monomeric species. This suggests the possibility that interconversion between monomeric and dimeric forms may be important for biological activity [12]. Co-IP studies have also revealed that the adenosine receptor A_2_AR, dopamine receptor D_2_R and mGluR_5_ receptors can form higher order oligomers [32], whereas the OX_1_ receptor can form homodimers and even higher order oligomers [23].

When this approach is used to study GPCR quaternary structure, the cells containing the putative oligomers must first be lysed and solubilized. An antibody raised against one of the receptors or against epitope sequences such as V5, His or Myc fused to one of the receptors is used to separate the oligomer from the mixture of other proteins present in the lysate by, for instance the addition of protein G or A sepharose. This binds the antibody and allows separation by centrifugation. The oligomer may then be resolved by SDS/PAGE and the second receptor detected by Western blotting with a second antibody raised against the second receptor or an epitope tag to which it is fused [33].

Co-IP can be effective for detecting GPCR dimers and oligomers, both homodimers/oligomers and heterodimers/oligomers. However, a significant restriction to the use of Co-IP in the study of oligmerization in native systems is the paucity of specific and high-affinity antibodies to GPCRs themselves. Therefore, the ability to differentially epitope-tag GPCRs has been central to the use of Co-IP, though this does tend to limit the use of the technique to heterologously expressed receptors [22]. A further drawback of this methodology is that the sample lysis and solubilization required for releasing the protein of interest from its insoluble membrane environment, is a step that may, by itself, lead to artificial protein–protein associations being formed or possibly destroying existing associations. However, as an early and classic method, the Co-IP plus Western blotting approach is still used for detecting receptor–receptor interactions *in vivo* [33].

## BN-PAGE

**Figure 2:**
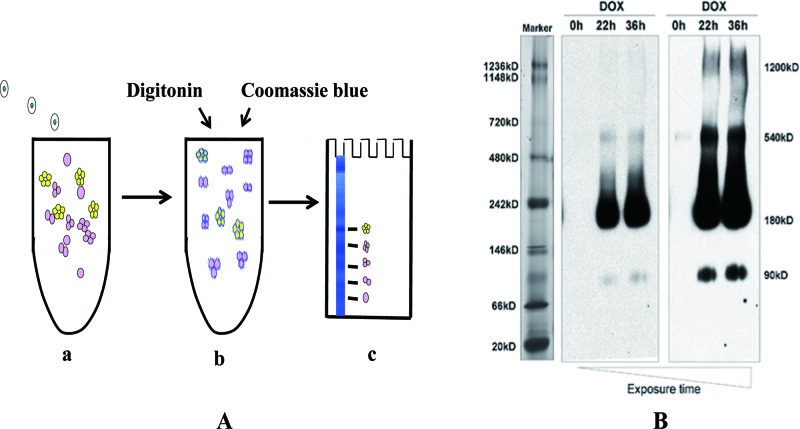
Principles of BN-PAGE (**A**) Cartoon representation of the major steps involved in BN-PAGE. (**i**) A mixture of proteins and protein complexes. (**ii**) Protein complexes are solubilized by mild non-ionic detergents (such as digitonin) and given a negative charge by adding Coomassie Blue that ‘coats’ the proteins in a similar manner to SDS, but without destroying quaternary structure. (**iii**) Proteins and complexes are separated by electrophoresis according to their molecular weight. (**B**) BN-PAGE shows that VSV-G–OX_1_–eYFP migrates consistently with a predominantly dimeric structure. Samples were transferred onto a PVDF membrane and immunoblotted to detect the VSV–G tag, with two different exposures of the same samples shown. The migration of protein molecular mass markers is shown in the left-hand lane [23].

BN-PAGE, developed by Schägger and von Jagow [34] was probably the first method to allow electrophoretic separation of intact respiratory protein complexes through the combined use of mild detergents and Coomassie Blue dye rather than the highly denaturing detergent SDS (Figure 2A). Thus, a gentle solubilization method yields protein complexes in their native states [35]. Although, Coomassie Blue is used because it binds to proteins non-specifically and gives them an overall negative charge, resulting in rapid electrophoretic mobility of the proteins towards the cathode at neutral pH [34]. Moreover, Coomassie Blue can prevent protein aggregation in the stacking gel during electrophoresis [15].

BN-PAGE has been shown to be useful to the study of the oligomeric state of proteins or protein complexes in plants [36,37]. The dimeric state of the muscarinic M_1_ receptor and the effect of MT7 toxin upon this state have also been analysed by this technique. The results indicated that the toxin either bound to and stabilized a dimeric form of the receptor or favoured the formation of the dimer [38]. Utilized as part of a wider study, Ward et al. [39] employed this technique as a proof of concept to demonstrate a change in the quaternary state of the epidermal growth factor receptor (EGFR) after ligand treatment. Fiala et al. [15,39] investigated multiprotein complexes (MPCs) by using BN-PAGE and showed that MPCs separated using BN-PAGE can be further subdivided into their individual constituents by using SDS/PAGE; the study revealed that BN-PAGE was suitable not only for identifying a specific MPC, but also for estimating the stoichiometry of the MPC constituents when performed as a native antibody-based mobility-shift assay [15].

An investigation was carried out by Xu et al. [23] into the quaternary structure of the OX_1_ receptor [23], this made use of BN-PAGE to determine the stoichiometry of the different forms of receptor oligomer. Proteins were solubilized with dodecylmaltoside and then subjected to BN-PAGE, the results of which indicated that the receptor constructs incorporating a YFP tag linked to the C-terminus or a SNAP tag at the N-terminus migrated predominantly as a dimeric species, though with additional bands indicating the presence of higher order complexes (Figure 2B). Treatment with SDS prior to BN-PAGE separation resulted in the detection of mostly dimeric species, with all of the higher order complexes being dissociated and migrating at the size predicted for the dimeric or monomeric species [23]. Native-bovine rhodopsin has been shown to exist mainly as a dimer and a higher order oligomer based on BN-PAGE and SDS/PAGE results used together with the findings from AFM and molecular modelling of the supramolecular structure and packing arrangement of murine rhodopsin dimers [14].

BN-PAGE is an easily accessible technique that performs a useful role in the study of GPCR quaternary structure and although not without drawbacks, it has enabled the successful determination of the proportions of different oligomeric species of a GPCR [23].

### Biophysical methods

#### FRET

**Figure 3:**
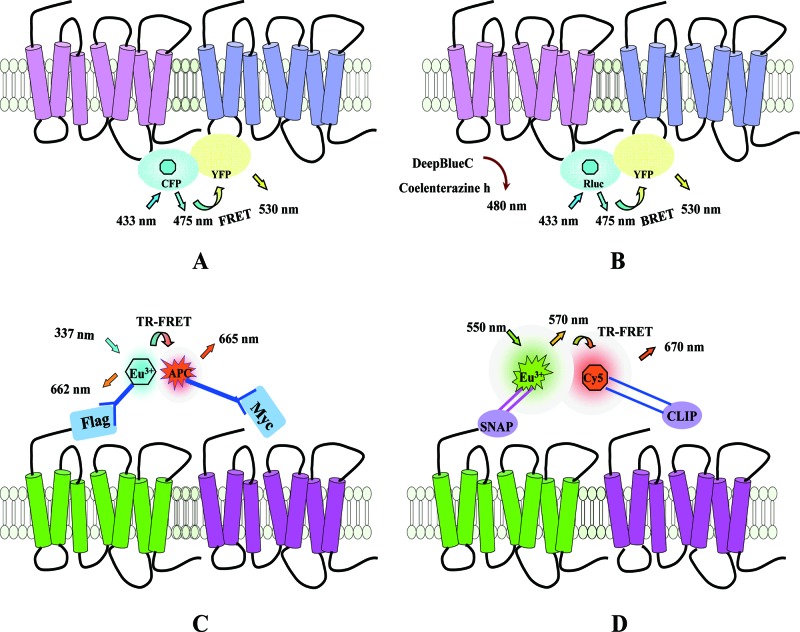
Principles of FRET, BRET and time-resolved FRET (TR-FRET) (**A**) The principle of FRET using CFP and YFP as a donor-acceptor pair. FRET occurs when the donor (CFP, shown in cyan) and the acceptor (YFP, shown in yellow) are in close proximity (<10 nm), so that upon excitation of CFP (with light at a wavelength of 433 nm), energy is transferred to YFP and causes emission (at 530 nm). (**B**) The principle of BRET is that energy transfer occurs between the luciferase and YFP. The reaction is initiated by addition of the substrate of luciferase, coelenterazine h, which results in the emission of light at 480 nm. If the distance between the donor and acceptor is <10 nm, energy is transferred from the luciferase to the YFP, resulting in a signal emitted by the YFP at 530 nm. (**C**) The principle of TR-FRET. A potential dimer pair of receptors bearing either Myc- or Flag-epitope tags is labelled with anti-Myc or -Flag antibodies against these. The antibodies are conjugated to either the ‘donor’ (Eu3+) species or the ‘acceptor’ (allophycocyanin, APC). (**D**) A potential dimer pair of receptors fused to SNAP and CLIP tags is treated with specific substrates for SNAP-tag and CLIP-tag, which have been linked to the ‘donor’ (Eu3+) and ‘acceptor’ (Cy5).

FRET is a non-invasive and non-destructive method for studying protein–protein interactions. The principle of resonance energy transfer was first illustrated in the late 1940s by Förster [40]. FRET is a physical phenomenon in which energy transfers from one excited fluorescent protein (the donor) to another (the acceptor) in a non-radiative (dipole–dipole) manner [41], assuming certain conditions that are required for FRET to occur are met [42,43]. One key parameter is that there must be an overlap between the emission spectrum of the donor molecule and the excitation spectrum of the acceptor molecule, whereas another is that the donor and the acceptor must be in close proximity, generally less than 100-Å apart [44]. GFP from the crystal jelly *Aequoreavictoria* and its variants such as CFP/YFP were the fluorophores most commonly used in early analyses for labelling potential ‘partner’ proteins of interest and we now refer to the use of these as classical FRET analyses (Figure 3A). Previously, in some applications, YFP has been replaced with a fluorescein arsenical hairpin binder (FlAsH) sequence, the binding of which by an inherently non-fluorescent ligand generates a fluorescent species that can function as an energy acceptor for the donor CFP [45,46].

FRET may form the basis of sensitive biosensors that can provide spatial and temporal information related to the dynamics of protein–protein interactions [47]. Intramolecular FRET sensors can detect GPCR activation in response to ligands [48] and ligand-induced GPCR signal transduction can also be detected [49]. In FRET studies of GPCR oligomerization, two different GFP variants acting as donor–acceptor fluorescent-protein pairs are typically attached to the same or different GPCRs and any interactions detected by a change in FRET maybe indicative of GPCR oligomerization [50,51]. By using this method, numerous GPCRs have been shown to exist or function as dimers or oligomers, for example mGluR functions as homodimers [52], whereas α1b-AR exists as higher order oligomers [53].

An important limitation of some techniques, FRET included, is that they cannot detect interactions occurring specifically at the membrane [44]. Additionally, GFP and its variants are sufficiently large to potentially affect protein functions [51] or interactions and furthermore such factors as cross-talk, bleed-through and photobleaching can influence accuracy [54]. However, FRET plays a crucial role in GPCR oligomerization analysis and has found a significant niche in the area of ligand screening and drug discovery.

#### BRET

BRET, which occurs when energy transfers from a luminescent donor to a fluorescent protein, was first used to study the interaction of circadian clock proteins in bacteria [55]. Subsequently, BRET has been used to investigate a variety of protein–protein interactions, including receptor–receptor interactions in living cells. Luciferase from *Renilla reniformis* (RLuc) is the most commonly used bioluminescent protein. Depending on the type of enzyme substrate and the nature of donor/acceptor pairs, BRET can be classified into different generations: BRET1 [56] (Figure 3B), BRET2 [56], BRET3 [57,58], eBRET [59] and QD-BRET [60].

BRET is widely used to investigate receptor interactions and has played a major role in the evolution of the GPCR oligomerization concept [61]. By using BRET, vasopressin and oxytocin receptors were found to assemble into oligomers early during their synthesis [62] and AT_1_Rs were shown to form heteromers with dopamine D_2_ receptors, an interaction that enables AT_1_R-selective drugs to alter the D_2_ receptor functional response [63]. The application of BRET to GPCR studies is not limited to the assessment of receptor oligomerization; it has also been used to assess GPCR interactions with other proteins and the characterization of GPCR activation and signalling [64]. BRET serves as a powerful tool for studying structure and function in GPCR–receptor tyrosine kinase (RTK) interactions and detecting the activation of various signalling pathways [56].

BRET signals cannot precisely distinguish cell surface-targeted receptors from receptors retained inside cells and so, when studying the receptors that are overexpressed, the accuracy of BRET may also be affected [57]. However, as a non-invasive technique that provides a high signal-to-noise ratio, BRET has been widely used in the study of GPCR oligomers.

#### TR-FRET

TR-FRET is a form of FRET based upon the use of a fluorescent donor incorporating lanthanide elements, for instance terbium or europium, whose most important characteristic is that they exhibit long-lasting fluorescent light emission [57]. The advantage of this is that a delay can be introduced after the initial excitation, but before the FRET signal is recorded, thus allowing other forms of biological fluorescence to decay prior to measurement. Thus, a significant improvement in signal-to-noise ratio is seen. Cryptates are the most common type of ‘cages’ developed as lanthanide carriers that can be used to label receptors of interest and they can markedly improve lanthanide properties [65]. In this system, energy transfers from the lanthanide to another fluorescent molecule such as Alexa Fluor 647 (A647) or APC [44]. The receptors can be labelled non-covalently (for instance with donor- and acceptor-labelled antibodies) or covalently using tag proteins such as SNAP, CLIP or Halotag. These are proteins, based upon O6-alkylguanine-DNA alkyltransferase (SNAP/CLIP) or haloalkane dehalogenase (Halotag), which have been modified to allow them to bind covalently to O6-benzylguanine (BG) (SNAP), O6-benzylcytosine (BC) (CLIP) or a chloroalkane (Halotag), compounds that are themselves linked to a variety of useful labels such as fluorophores or the htrFRET reagents. Thus, a potential dimer pair of receptors can be fused to SNAP and CLIP respectively, expressed in cells and treated with TR-FRET donor and acceptor substrates for the SNAP or CLIP tags (Figure 3C, D). If the tags are in close proximity that is if the receptors form a dimer, a TR-FRET signal will be generated. Hence, by non-covalent and covalent fluorophore labelling of the protein of interest, it is possible to study interaction of receptors at the plasma membrane.

Albizu et al. [66] validated the TR-FRET strategy and showed that the peptide receptors vasopressin V1a, V2, oxytocin [57,62,67] and a biogenic-amine receptor (dopamine D_2_) [68–71] can dimerize. Furthermore, it was shown for the first time that oxytocin receptors form dimers in native tissues. By using TR-FRET, GABAB receptors were confirmed to form not only dimers but also higher order oligomers, in which the GABAB1 receptor might mediate the interaction, whereas mGluRs were found to assemble into strict dimers [72], although human M_3_ receptors were shown to exist as dimeric/oligomeric complexes that can be modified by ligand binding [73]. Moreover, homogenous TR-FRET studies revealed the coexistence of functional homomers and heteromers of dopamine D_2L_ and D_3_ receptors at the cell surface [74].

A clear advantage of TR-FRET is its high signal-to-noise ratio, which is due to several factors. Firstly, TR-FRET can distinguish between long-lived and short-lived fluorescence and complete its temporal selection. Secondly, because lanthanide emission is not polarized, TR-FRET is mostly independent of fluorophore orientation. Thirdly, europium and terbium exhibit complex emission spectra featuring multiple fluorescence peaks [75], which makes TR-FRET compatible with red Cy5- or dy647-like fluorophores and fluorescein-like fluorophores when used as acceptors. Reduced bleed-through is also an advantage [76].

#### FRAP

FRAP is an approach widely used for studying the diffusion of fluorescently labelled molecules, usually within membranes, often involving the investigation of the dynamics of GFP-tagged proteins in single cells by using confocal microscopy [77,78]. FRAP is a process in which fluorescently labelled molecules diffuse into a defined region of interest, whose molecules have been photobleached by the application of an intense pulse of laser light. The exchange rate, translational diffusion coefficient (*D*) and the mobile fraction (Mf) provide critical information regarding the properties of the molecules studied [79].

FRAP, alone or combined with other techniques, is widely used in GPCR studies and plays a significant role in GPCR drug discovery [80]. By using FRAP, both β_1_-AR and β_2_-AR were shown to be capable of forming oligomers [81] and real-time interactions between bradykinin type-2 receptor and β-arrestin-2 were detected [82]. Furthermore, FRAP analysis was used to examine how the actin cytoskeleton affects the mobility of the serotonin (5-hydroxytryptamine, 5-HT) 1A (5-HT_1A_) receptor and the implications that this has for signalling [83]. When combined with other techniques, a donor-FRAP approach could serve as a useful technique for studying protein–protein interactions, even interactions among more than two proteins [80]. By using a combination of FRAP with bimolecular fluorescence complementation (BiFC), heterodimers of the MOR and neuropeptide FF receptor 2 were shown for the first time to adopt a specific diffusion behaviour corresponding to a mixture of the dynamic properties of both receptors [84]. Moreover, the combined use of FRAP and live-cell imaging revealed the functional relationship among frizzled 6, disheveled and heterotrimeric G proteins [85]. Similarly, FRAP was combined with adhesion assays to elucidate the functional role of the domains of the chemokine CX3CL1 [86].

FRAP in lipidic cubic phase (LCP-FRAP) may be used as a pre-crystallization screening assay for membrane proteins, where it serves as a technique for the rapid identification of GPCR crystallization [87]. The data collection and processing protocols used with β_2_-AR and A_2_AR to map conditions that support adequate diffusion in LCP have now been validated [88].

#### PCAs

**Figure 4:**
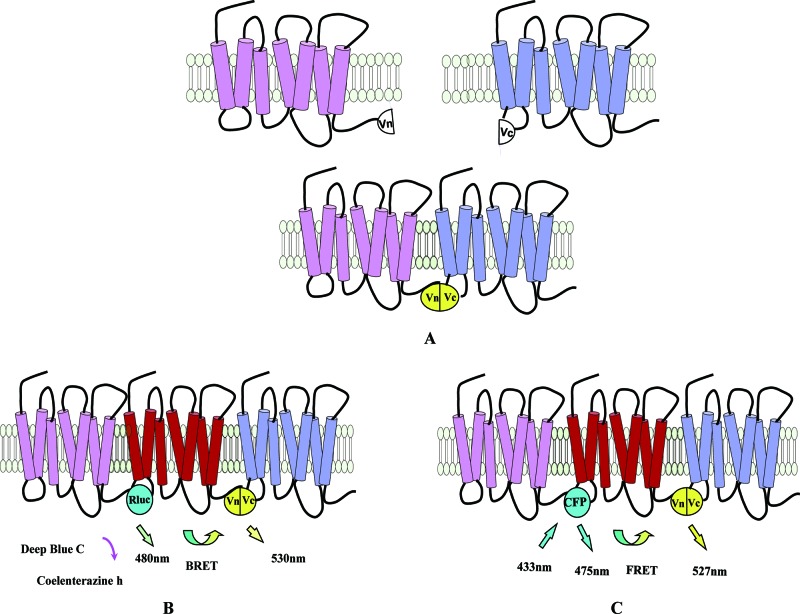
Schematic representation of PCA technology (**A**) Cartoon representation of PCA. When the two receptors fused to two complementary fluorescent protein fragments (Vn and Vc) are in close proximity, fluorescence can be detected. (**B**) When three receptors labelled with RLuc, Vn and Vc are in close proximity, BRET will occur between RLuc and the re-assembled fluorescent protein. (**C**) FRET can be detected when three receptors fused to CFP, Vn and Vc are in close proximity.

Fluorescent and bioluminescent PCAs, also known as BiFC and bimolecular luminescence complementation (BiLC) assays respectively, have recently been used to investigate GPCR interactions [89]. The idea behind this is that when two receptors fused to two protein fragments that are complementary parts of a single fluorescent/bioluminescent protein are in close proximity, such as when the receptors form a dimer, the fragments form a functional protein and fluorescence/bioluminescence can be detected [89] (Figure 4).

Fluorescent or bioluminescent proteins used in PCAs include luciferases from *Renilla reniformis* and *Gaussia princeps* (RLuc and GLuc) [90] and fluorescent proteins, some of which have been split successfully [91] such as Venus [92], Cerulean [93] and mCherry [94].

By using BiFC, the existence of mGluR_5_ and D_2_R homodimers in living cells was visualized for the first time [95]. When combined with approaches such as FRET and BRET, BiFC and BiLC allowed the detection of multiple protein interactions. For example, BiFC-BiLC assays were used to identify the tetrameric complexes of β_2_-AR and D_2_R [96] and tetramers of D_2_R [97]. BiFC-BRET was used to demonstrate the existence of adenosine A_2_AR tetramers [98] whose accumulation at the plasma membrane in differentiated neuronal cells was also detected using combined BiFC-FRET techniques [99].

The interaction between A_2_AR and D_2_R was examined using BiFC [100]. Multicolour BiFC assays were used to measure heteromeric A_2_AR–D_2_R and homomeric receptor complexes (A_2__A_R–A_2A_R or D_2_R–D_2_R) at the cell surface and in intracellular compartments [101]. This method was also used to detect the existence of CB_1_R and D_2_R heterodimers and their subcellular localization and regulation processes [102]. Consistent with the results of other biochemical and behavioural studies [103], BiFC-BRET data support the existence of A_2A_R, CB_1_R and D_2_R complexes [104] that can associate with other GPCRs. For example higher order oligomers of A_2A_R, D_2_R and mGluR_5_ were detected using BiFC-BRET and sequential resonance energy transfer in live cells, which agreed with the findings of a Co-IP analysis [95].

BiFC and BiLC can detect subcellular interactions of GPCRs and only require basic experiment methods when compared with other approaches [89]. However, a potential drawback of PCA-based assays is that the interaction among complementary protein fragments might affect the function or localization of the receptors. It may even be that interactions of the complementary fragments could lead to the detection of protein–protein interactions that have been forced to occur by the process of bimolecular complementation [89,91].

#### FRET spectrometry

FRET spectrometry is an experimental approach used to determine the stoichiometry and quaternary structure of proteins. It is based on FRET and uses optical microspectroscopy technology in order to probe the structure of dynamic protein complexes in living cells [105].

FRET spectrometry depends on measuring and analysing the distributions of apparent FRET efficiency (Eapp), across FRET-image pixels of individual cells expressing proteins of interest [106]. Eapp relies on only one parameter, the pairwise FRET efficiency (Ep), which is the efficiency of energy transfer between a single donor and a single acceptor [105]. The Eapp distribution features several peaks that together constitute a unique FRET spectrum of distinct oligomers corresponding to the configuration structure and from the number of these peaks, the most likely quaternary structure of a complex can be identified [107]. The model that produces the closest agreement between the peak positions of simulated and experimental Eapp distributions is considered the quaternary structure of the protein. Because such peaks collectively create a unique FRET spectrum in which each peak corresponds to an oligomeric configuration of the protein, this could be called a FRET spectrum. Thus, FRET here becomes a spectrometric method, hence FRET spectrometry, for sorting protein complexes according to their size and shape [105].

**Figure 5:**
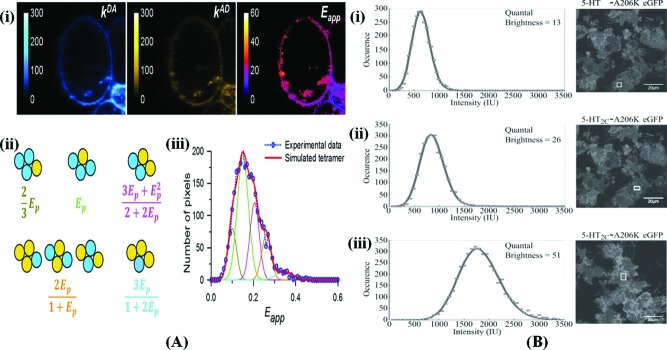
The application of FRET spectrometry to the study of GPCR oligomerization (**A**) FRET spectrometry analysis of Flp-In TM T-REx TM-293 cells expressing Myc–Y149C/A239GM_3_R–Cerulean (inducible) and FLAG–M_3_R–Citrine (constitutive). (**i**) Photomicrograph of a cell showing donor fluorescence in the presence of acceptor, acceptor fluorescence in the presence of donor and the Eapp distribution map at each pixel. (**ii**) Distinct configurations of donors and acceptors within a parallelogram (or rhombus)-shaped tetramer and their Eapp. (**iii**) Eapp histograms obtained from pixels representing the plasma membrane of the cell shown in (i) and the theoretical best fit with a sum of five correlated Gaussian peaks whose positions are given by a single adjustable parameter Ep (=0.18) via the rhombus tetramer model shown in (ii) (from Patowary et al. [107]). (**B**) Quantal brightness analysis in the technique of SpIDA of 5-HT_2C_. Each intensity histogram was fit to a single population model with results consistent with the receptor being a monomer, a dimer and a tetramer [39].

FRET spectrometry has been used to determine the number and the relative disposition of protomers within homooligomers of a model GPCR, the sterile-2a factor receptor (Ste2p) from the yeast *Saccharomyces cerevisiae*; a model of a tetramer configured as a rhombus provided the best fit [106]. FRET spectrometry has been used to investigate the formation of muscarinic M_3_-receptor oligomers at the plasma membrane. By tightly controlling the ratio of the concentrations of donors and acceptors expressed by the cells, it was determined that M_3_Rs at the plasma membrane exist as stable dimeric complexes and as tetramers that can dynamically interconvert with dimers [107] (Figure 5A).

In order to carry out FRET spectrometry, FRET spectrograms must be acquired and a fluorescence-imaging method must be used that gives the required molecular resolution and provides FRET efficiency values at each image pixel. The FRET spectrometry method performs best when the expression level of protein complexes with stable structures is so low that one voxel in the sample space contains a single protein complex [105].

### Physiological methods

#### SpIDA

SpIDA is an image-analysis technique that was inspired by the temporal photon counting histogram approach, which can be used to measure fluorescent-macromolecule densities and oligomerization states sampled from within individual laser scanning confocal images. The method is based on fitting super-Poissonian distributions to the intensity histograms calculated from selected regions of interest within the images to obtain density values for fluorescent molecules and also their quantal brightness [108]. The intensity histograms are plotted from the number of pixels for each intensity value within a defined region of interest. This intensity value is the integrated fluorescence from within the beam focal volume at a given position. Histogram fitting functions are then calculated by computing the fluorescence intensity of all possible configurations of particles in the beam focal volume, following which the values obtained for each of these possible particle configurations are weighed according to their probability [109], as in the case of photon counting [110]. In accordance with FRET findings [111], SpIDA results have demonstrated that the EGFR can form EGF-induced dimers and further revealed the dynamic changes that occur in receptor oligomerization [111,112]. Moreover, SpIDA results successfully confirmed the existence of GABAB oligomers [113]. SpIDA has been used to investigate the regulation of 5-HT_2C_ receptor oligomerization and identified the presence of multiple forms ranging from monomers to higher order oligomers [39] (Figure 5B).

SpIDA has enabled quantification of protein interactions in native tissues by using standard fluorescence microscopy [111]. In conclusion, SpIDA allows detection of homooligomerization and facilitates dynamic studies on trafficking and molecular interactions in live cells and can also yield information on monomer-to-dimer ratios [110].

#### Single-molecule imaging

Single fluorescent-molecule video imaging appears to be a highly suitable method for determining both whether GPCRs form dimers or oligomers and how long these forms last. As a kind of single fluorescent-molecule imaging method, TIRFM has demonstrated that β_1_-ARs, β_2_-ARs and GABAB receptors can form dimers and oligomers and have revealed their spatio-temporal characteristics at single-molecule resolution. By using these techniques, all fluorescent GPCR molecules at the cell surface could be tracked and the findings obtained may have important implications for our views of the interactions of GPCRs with each other and with other signalling molecules [114]. In 2010, Hern et al. [115] reported the first results of GPCR single-molecule imaging study, which showed that the dimer lifetime of M_1_ muscarinic receptors was 0.7 s (23ºC) [115,116]. In 2011, for the first time, Kasai et al. [117] put forward a single molecule methodology, named superquantification.

In single fluorescent-molecule imaging, the first step is the labelling of the protein of interest and for this purpose, fluorescent proteins like GFP and its variants are rarely used. Kasai et al. [117] labelled the *N*-formyl peptide receptor (FPR), a chemoattractant class A GPCR, with the agonist formyl peptide conjugated with the fluorescent dye Alexa Fluor 594 at exactly a 1:1 stoichiometric ratio at 37ºC. Using TIRF illumination [115], the process by which ligands bind to the FPR molecules located at the basolateral cell membrane was observed at the single-molecule level. Based on the single-molecule images, a 2D–3D Scatchard plot was constructed and used to determine the number of FPR molecules bound by the fluorescent ligand, the dynamic equilibrium of the monomer and dimers, the 2D equilibrium constant and the dissociation and association rate constants [114,117]. Single-molecule analysis has shown that β_1_-AR and β_2_-AR form transient homodimers (heterodimers were not examined) exhibiting a lifetime of 4 s at 20.5ºC , which is approximately 40 times longer than that of FPR (37.8ºC) and six times longer than that of M_1_ receptor (23.8ºC) [118].

Although the signal-to-noise ratio of the observed single-molecule spots and the fluctuation of the background are the main challenges of this technique [114], single-molecule imaging maybe expected to contribute substantially to the field of cell biology and in the study of molecular interactions.

#### X-ray crystallography of GPCRs

The determination of GPCR structure at high resolution using X-ray crystallography began with the solution of the structure of rhodopsin [119], whose first crystal structure was solved from diffraction data extending to 2.8-Å resolution. There then followed a lengthy period during which no GPCR structures were published until the work of Park et al. solved the structure of opsin [120]. The explanation for this period of dearth of X-ray crystallographic GPCR data is that it is due to problems related to their expression, purification, intrinsic chemical heterogeneity and instability. These were, however, overcome and subsequently an increasing number of X-ray crystallographic GPCR structures have appeared [121–123]. The relevance of crystal structures to GPCR oligomerization is the suggestion that the proteins will crystallize utilizing the same intermolecular contacts and faces as can be found when diffusing through the cell membrane and indeed several distinct potential dimer interfaces are starting to emerge from crystallographic studies. The first semi-empirical model of a higher order crystal structure of rhodopsin and opsin (PDB: 1N3M) in native membranes identified two interfaces, TM4/TM5 and TM1/TM2-CL3 (cytoplasmic loop connecting helices V and VI) [124]. The first X-ray crystal structure of a ligand-free basal-state receptor was the structure of oligomeric turkey β_1_-AR (PDB: 4GPO), which also displayed two alternating dimer interfaces, involving TM4/TM5 and TM1/TM2/H8 [29]. The structures of both the ground state and a photoactivated, deprotonated intermediate of bovine rhodopsin at 4.15-Å resolution have been reported, which revealed that these receptors form a potentially physiologically relevant dimer interface involving TM1, TM2 and TM8 [125]; this is similar to that shown by the 2.9-Å crystal structure of human κ-opioid receptor (PDB: 4DJH) in complex with the selective antagonist JDTic ((3R)-7-hydroxy-N-[(1S)-1-(((3R,4R)-4-(3-hydroxyphenyl)-3,4-dimethyl-1-piperidinyl)methyl)-2-methylpropyl]-1,2,3,4-tetrahydro-3-isoquinoline-carboxamide), which revealed an arrangement of parallel dimers [123]. The 2.8-Å crystal structure of the MOR (PDB: 4DKL) in complex with an irreversible antagonist showed that the receptor crystallizes as a two-fold symmetrical dimer through a four-helix-bundle motif formed by TM5 and TM6 [28]. Moreover, five independent crystal structures of CXCR4 (PDB: 3ODU) in complex with antagonists at 2.5 to 3.2 Å resolution showed that the homodimers in crystals share the same interfaces [121]. Metarhodopsin II (PDB: 3PXO) structures, show two openings of the retinal-binding pocket, one between TM5 and TM6, which arises mainly from side-chain changes of Phe-208^5.43^, Phe-212^5.47^ and Phe-273^6.56^, which are also observed in the Ops*structure 6, whereas the other, which is between TM1 and TM7, is caused by a rotamer change of Phe-293^7.40^ relative to rhodopsin [122]. Furthermore, the histamine H_1_ receptor structure displays one ligand pocket beside the TM3/TM4/TM5 interface, which is a strictly conserved residue in aminergic receptors and forms an anchor salt bridge with the amine moiety of the ligand and another near the TM4/TM4 interface [126].

Crystallography can contribute to our knowledge of GPCR dimerization and oligomerization and may provide information regarding receptor interfaces, although care must be exercised as some of these interfaces might be artificial and potentially may not represent a functional biological assembly. However, the findings still suggest possible scenarios related to the manner in which GPCRs interact with each other and possibly the mechanisms of GPCR function.

#### Computational methods

A considerable amount of experimental evidence has demonstrated that GPCRs function either as dimers or higher order oligomers. However, it is challenging to use experimental approaches for constructing structural models of dimeric or oligomeric GPCRs at a detailed molecular level. Structural approaches such as cryo-EM and AFM were used to obtain low-resolution oligomeric-structure information about GPCRs [127,128] and by using crystallography, GPCR monomer and oligomer structures were obtained. Determination of the GPCR structures facilitated structure-based prediction of GPCR dimerization by using computational methods such as protein–protein docking, molecular dynamics (MD) simulation and coarse-grained MD (CGMD) simulation.

### Structure-based approaches

#### Protein–protein docking

Protein–protein docking algorithms, the most commonly used tools for modelling GPCR oligomeric structures [129], mainly consider the atomic coordinates of two interacting proteins as rigid bodies and produce a number of plausible solutions. The method can provide, quickly and at low computational cost, possible dimer configurations, which can then be either used to design experiments to test the possible dimer configurations [130].

Among all the docking programs tested by Kaczor et al. [131] (ZDOCK, ClusPro, HEX, GRAMM-X, PatchDock, SymmDock, HADDOCK, 3D-GARDEN and ROSETTA), GRAMM-X performs optimally in modelling TM complexes [132]. This program was used to model the 5-HT_4_ receptor homodimer, which identified TM2/4 and TM4/6 as the potential dimer interfaces [133]. Fanelli et al. [134] applied a combination of ZDOCK and subsequent membrane filters to the homodimer of the thromboxane A_2_ receptor (TXA_2_R), isoforma and predicted that TM1 mediated the intermolecular contacts for both protomers of the best predicted homodimer.

As compared with most sequence-based methods, protein–protein docking can generate dimer configurations more quickly and readily and also yield the coordinates of entire complexes from basic physical principles [129]. However, as a type of computational high-throughput method, this approach can only provide static images because of the rigid modelling [135].

#### All-atom MD simulations

All-atom MD simulations are established based upon experimental data. Given the availability of GPCR crystal structures in particular, the approach has been widely used to describe the dynamic behaviour of these receptors at an atomic level.

Based on the AFM-derived structure of rhodopsin dimers presenting the TM4/5 interface [124], Neri et al. [136] suggested an asymmetric signal transduction mechanism through the prototypical GPCR rhodopsin dimer by using an extensive MD simulation and found an interface-mediated pathway of the dimerization. This suggests that oligomerization-aided signal transduction maybe a biological strategy to enhance activation efficiency across the whole family of GPCRs [136]. Filizola et al. [137] conducted a 45-ns all-atom MD simulation and determined that the asymmetrical dynamic properties might be relevant to a possible asymmetric activation mechanism of GPCRs, which agreed with the finding of Cordomí and Perez [138], who developed a 100-ns MD study. Moreover, a 40-ns MD simulation was performed on the heterodimeric complex of the mGluR_2_ and 5-HT_2A_ receptors, which was also assembled around the TM4/5 interface [139]. Relying on an interface proposed by Schultz et al. [140] for the vasopressin receptor V2R dimer, a V_2_R tetramer was studied by a 5-ns all-atom MD simulation, which indicated that TM3/4–TM6/7 interface relates to the transactivation between active and inactive monomers [141]. Furthermore, MD simulations were performed on four different MOR–DOR heterodimers and from that TM1/7 and TM4/5 were identified as the most likely interfaces [142].

Compared with other methods, MD simulations generate images featuring higher spatial resolution over a longer timescale, which may reflect the dynamic properties of dimers [143]. However, the MD timescale is nanoseconds to microseconds, which can reflect only the minor conformational changes of GPCRs. In addition to this, the construction of a GPCR dimer–membrane complex prior to the MD simulation is a challenging task [129].

#### CGMD simulation

The CGMD technique enables, simulations to be performed on larger systems and for longer times when compared with all-atom MD, but at the cost of lower resolution and accuracy [144].

In the analysis of the DOR performed using CGMD and umbrella-sampling simulation by which theoretical values of the thermodynamics and kinetics of the DOR dimerization were derived, TM4 was found to be critical for dimer formation [145], indeed, more important than TM4/5 [146]. Periole et al. [147] used large-scale CGMD to study the self-assembly behaviour of rhodopsin and found that TM4, TM5 and TM6 may form interfaces related to high-order oligomerization of rhodopsin. Mondal et al. [148] determined that the oligomerization of β_2_-AR at specific interfaces involves TM1, TM4 and TM5, whereas in other studies, TM1/TM1 and H8/H8 were found to form the most stable dimerization interfaces [146,149,150].

The CGMD strategy can be used to study the homo- and heterodimerization of GPCRs and other TM proteins, which may deepen our understanding of the forces involved in the membrane organization of integral membrane proteins [147].

#### Two-dimensional Brownian dynamics simulation

The 2D Brownian dynamics (2D-BD) simulation approach is used to predict protein–protein interactions [151]. The technique is based on the theory that the movement of membrane proteins is restricted in the nearly planar environment of the membrane (2D). To predict dimerization of membrane proteins, the BD program (MacroDox) is adapted to include both a hybrid electrostatic potential map of membrane and water for electrostatic interaction calculations and post-docking refinement for protein flexibility [152]. The 2D-BD approach has been used to predict several protein complexes whose individual crystal structures are available and was tested successfully by performing a re-docking simulation experiment on the β_1_-AR oligomeric-form crystal structure (PDB: 4GPO) [29]. The technique was successfully used to predict the dimerization of outer-membrane phospholipase A and glycophorin A [153].

#### Sequence-based approaches

Here, we describe bioinformatics techniques that are based upon protein (primary) sequence rather than obtaining information from crystal structures and other experiment approaches. These sequence-based techniques can be used to create models of monomers, dimers and oligomers of GPCRs and even identify dimer interfaces of GPCRs [129]. This technique can be divided into two types. One presumes that the interface of all subtypes is conserved among subfamilies, whereas the other supposes that the interface can change among subtypes of the same subfamily [154]. Here, we give an introduction to this type of approach.

#### Evolutionary trace method

The evolutionary trace (ET) method, which was described by Lichtarge et al. [155,156] is a technique used to determine functional sites in a protein whose 3D structure and sequence alignment are known. Based on the idea that proteins which evolved from a common ancestor would have a similar backbone structure [157], the ET approach hypothesizes that the protein family retains its folding in the sequence alignment. It also presumes that the protein family should conserve the location of functional sites and have a distinctly lower mutation rate at these sites.

Using over 700 aligned GPCR sequences, an enhanced ET method using Monte Carlo techniques [158] suggested that a major potential functional site on the lipid-exposed faces of TM5 and TM6 is common to each family or subfamily of receptors. In addition, a second functional site was identified on TM2 and TM3 that was predicted to function in the formation of either heterodimers or higher order oligomers, whereas TM1, TM4 and TM7 were suggested to have less functionality [159]. The ET method was also applied to a multiple-sequence alignment of visual opsin, bioamine, olfactory and chemokine class A GPCRs with the result that clusters of residues responsible for global and class-specific functions have been identified [160].

#### Subtractive correlated mutation analysis

Subtractive correlated mutation (SCM) analyses have been improved over correlated mutation analysis (CMA) by using filtering algorithms [161]. The sequences of two proteins from the same organism, are merged at first and SCM is applied to the merged sequences. By removing the intramolecular correlated residues in both monomers from all the correlated residue pairs identified from the merged sequences, the interface-forming residues of two proteins can be found [129]. Using this method, TM4, TM5 and TM6 of DOR and TM1 of MOR were shown to be involved in the formation of heterodimer interfaces [161].

#### Hidden-site class model of evolution

Evolutionary relationships among proteins can generally be represented by a matrix indicating the rate at which every amino acid substitution occurs during evolution. Most methods for studying protein–protein interactions that involve evolution use a single substitution matrix for all locations in all protein sequences, which is a limitation because an amino acid substitution at a particular location in the sequence of a protein will not always produce the same functional effects. The hidden-site class model applies different substitution matrices to represent amino acid substitutions at distinct locations in a protein sequence [162]. This method has been shown to give improved phylogenetic inferences by identifying locations in the sequences considered to be under similar selective pressure and by characterizing changes in selective pressure. Locations that are assigned to site classes exhibiting the lowest rate of substitution are expected to correspond to structurally or functionally critical positions. Soyer et al. [163] used this model to perform a family-specific analysis of GPCRs that included 199 class A GPCRs and identified lipid-exposed evolutionarily conserved locations on TM4, TM5 and TM6 in different subfamilies.

#### Spatial cluster detection

Spatial cluster detection (SCD) is a method involving the two problems, one is the subtype specificity of the interface and the evaluation of the spatial location of the detected residues [164]. In an advanced approach, a 3D structure was assumed to be a thick tube and was reduced to a 2D ring-like structure by projecting all residues on a plane perpendicular to the tube axis. Interface residues were then expected to cluster in a sector of this 2D ring-like structure. SCD was applied to several GPCR subtypes, such as dopaminergic and ARs, muscarinic acetylcholine receptors and opsin-family receptors. Notably, TM4 and TM6 were found to be involved in the interfaces of D_2_R and β_2_-AR respectively, which is in agreement with experimental data [12,165–167]. SCD-based predicted interfaces of various GPCRs are available online at the GRIP server; http://grip.cbrc.jp/GRIP/ [129].

## Concluding remarks

The fact that isolated monomeric seven TM domains of GPCRs are able to activate G-proteins when they themselves are activated by synthetic small molecules [168] is a great support to the idea that at least some monomeric GPCR units are functional. However, although some GPCRs can function as strict monomers, there is steadily accumulating evidence that they can exist and function as dimers or higher order oligomers in living cells [169].

A diverse range of approaches have led to a general appreciation that, at least some, GPCRs can exist as dimers, higher order oligomers or a mixture of these states and that such interactions may be important for the trafficking of GPCRs and their functions. Biochemical methods such as Co-IP and Western blotting serve as a useful starting point for verifying that GPCRs can form oligomeric complexes. BN-PAGE may confirm the existence of multiple oligomeric forms of a GPCR, whereas the use of biophysical techniques such as FRET, BRET and PCA is able to provide much vital data on GPCR quaternary structure. However, these techniques cannot be readily used to answer questions regarding the size of the oligomeric complexes and their potential dynamic nature. TIRFM and single-molecule imaging enable tracking of the position of single GPCRs and provide some information regarding the oligomeric state. The recent solution of several GPCR crystal structures and the development of computational approaches have provided further data to help answering questions regarding GPCR oligomerization. When used in combination with various experimental methods described above, these new approaches could be useful techniques for predicting the dimerization and oligomerization interfaces of GPCRs.

Much available evidence indicates that many members of GPCRs can form homomers and heteromers. Homodimers appear to be the predominant species and some may be able to form higher order oligomers, particularly tetramers. GPCR heteromers may serve as promising new drug-development targets. The search for receptor-heteromer-selective compounds is an interesting development that has emerged because of the continued discovery of functionally significant receptor heteromers [170]. Compounds or reagents that specifically target the heteromer interface are likely to not only serve as tools for exploring the physiological significance of GPCR heteromers: they could also lead to the development of highly selective drugs [169].

In summary, GPCR oligomers are involved in the regulation of numerous biological processes [1] and studies of these receptors may lead to the development of ligands exhibiting selective affinity and function.

## Abbreviations

APC: allophycocyanin
AR: adrenergic receptor
AT_1_R: angiotensin II receptor type 1
β_2_-AR: β_2_-adrenergic receptor
BiFC: bimolecular fluorescence complementation
BiLC: bimolecular luminescence complementation
BN-PAGE: blue-native PAGE
BRET: bioluminescence resonance energy transfer
CB_1_R: cannabinoid receptor type 1
CGMD: coarse-grained MD
Co-IP: co-immunoprecipitation
DOR: δ-opioid receptor
Eapp: apparent FRET efficiency
EGFR: epidermal growth factor receptor
Ep: pairwise FRET efficiency
ET: evolutionary trace
FPR: *N*-formyl peptide receptor
GABAB: γ-aminobutyric acid
GPCR: G-protein-coupled receptor
LCP: lipidic cubic phase
M_3_R: M_3_ muscarinic acetylcholine receptor
MC_4_R: melanocortin-4 receptor
MD: molecular dynamics
mGluR: metabotropic glutamate receptor
MOR: μ-opioid receptor
MPC: multiprotein complex
PCA: protein-fragment complementation assay
RLuc: luciferase from *Renilla reniformis*
SCD: spatial cluster detection
SCM: subtractive correlated mutation
SpIDA: spatial intensity distribution analysis
TIRFM: total internal reflection fluorescence microscopy
TM: transmembrane
TR-FRET: time-resolved FRET
2D-BD: 2D Brownian dynamics
5-HT: 5-hydroxytryptamine
